# Undertaking a face to face Summative OSCE assessment during the COVID-19 Pandemic - a descriptive narrative

**DOI:** 10.15694/mep.2020.000244.2

**Published:** 2021-03-17

**Authors:** Claire Darling-Pomranz, James Gray, Charlotte Watson

**Affiliations:** 1 University of Sheffield

**Keywords:** OSCE, Clinical Examination, Pandemic, COVID-19, Performance Assessment, Quality Assurance

## Abstract

This article was migrated. The article was marked as recommended.

The 2020 Covid19 pandemic has caused significant disruption to medical education across the world. At the University of Sheffield final year medical students undertook a virtual OSCE via technology in order to graduate however for our Physician Associate course this was more problematic as following completion of the assessments at the university students are also required to pass a national examination with a formal Objective Structured Clinical Examination (OSCE). Due to this it was felt that it was crucial to run an OSCE examination for our students on a face to face basis but within the context of managing the potential safety and risks inherent in doing so.

This piece of work describes the process of running the examination including detail which we feel will be useful to others who may seek to undertake examinations for the same reason within the current health emergency or any future such events. It is important to note that some innovations we put in place with respect to technology enhancing safety will remain for future OSCE examinations in any circumstances.

## Introduction

The Covid19 pandemic of 2020 has led to rapid changes in the way assessments have been carried out with a significant switch to online assessment methodologies. Whilst this provides us with a good alternative for traditional written assessments the provision of objective structured clinical examinations (OSCE) is much more problematic. The University of Sheffield Physician Associate course delivers a summative OSCE to between 16 and 22 students at the end of the second year of the course. This sitting involved 17 students. This is designed as both a summative course assessment and an opportunity to prepare students for the national OSCE assessment which they must pass in order to be permitted to enter the workforce. This made it important that, if possible, we delivered this examination as close to normal as possible to adequately prepare our students. This paper describes the steps we took in order to enable delivery of the OSCE whilst restrictions still applied during the Covid19 pandemic. We believe the steps taken and lessons learned can be applied to anyone seeking to run an OSCE examination in similar situations and builds on previous work undertaken by colleagues in Singapore ( Boursicot, *et al.* 2020).

## Initial planning

In order to undertake this examination we needed to ensure that the initial planning phase allowed us to reassure both students and our university that we could maintain a Covid secure environment for the examination. This is in line with guidance from the UK Health and Safety executive ( Health and Safety Executive, 2020).

Firstly we needed to identify an appropriate space which would allow the circuit to be suitably spread out permitting each individual station to use a larger footprint than normal to enable social distancing. During the planning stage the United Kingdom guidance changed from two metres to one metre with mitigations such as face coverings ( UK Cabinet Office, 2020) being in place. Use of such coverings risked presenting a barrier to students in being able to pick up cues effectively and actors to respond to them hence two metres was maintained as our standard. Face shields were considered but it was decided that as two metres could be maintained these would be available for any actors or students with particular concerns but otherwise not required. This would have been our mitigation of choice had two metre distancing been impossible due to constraints on building space. In space planning we also needed to identify sufficiently sized rooms to quarantine students as well as breakout spaces for participants to eat lunch, something we felt important for their general wellbeing during the day, rather than remaining on station.

Secondly risk assessments needed to be completed both for the process itself and for the individual students to ensure that no individual who might be considered higher risk would be unnecessarily exposed by undertaking this assessment. Student risk was assessed using a risk assessment tool developed by the Medical Schools Council with the overall risk assessment undertaken using a standard five by five (likelihood and severity) risk assessment tool. The risk assessment process also included mitigations so that no individual student was potentially disadvantaged due to being unable to participate in the assessment itself.

One key learning point was regarding assumptions on building usage. Many university buildings had been closed for three months and thus needed full health and safety checks including electrics and legionella screening. These checks took time to implement and obtain results for and could have led to potential delay, or inability to undertake the examination. We took the decision to move our original planned date by five days so that if testing found issues we could move to an alternative, but less ideal, building and run the circuit. Students were understanding of this decision.

## Circuit planning

When designing the OSCE circuit we needed to consider the type and number of stations to minimise the risk to any individuals involved and to enable sufficient space to maintain appropriate social distancing within stations. The original OSCE plan contained 14 stations in line with the UK National Physician Associate OSCE ( Royal College of Physicians, 2020). The decision was taken to reduce the number stations to ten and to remove procedures. Procedures had been assessed in our clinical skills training programme and in practice during clinical placement with all students having completed those satisfactorily. Discussion with our psychometric team confirmed that a minimum of nine stations was required in order for the circuit to ensure appropriate discrimination for this high stakes examination, ten ensured that we had some resilience should a station fail to perform as expected.

Of our ten stations, seven were considered to be communication stations, involving either information gathering or delivery, although one of these was an emergency scenario. These stations were simple in that all participants could be socially distanced at the necessary two metres presenting minimal risk to all. Three stations were examination stations and these were planned around the ability to utilise alternatives to traditional direct “hands-on” patient contact. One station was a mental health examination which therefore was manageable as for the communication stations, one station was a brief history and rectal examination which could be done using a simulation rig, and the final examination was an abdominal exam which could be performed on a simulation manikin with the examiner providing findings as the student went through the examination process. As the rectal examination rig and manikin were fully cleanable this allowed examinations to be assessed without risk to individuals.

The layout and timings for the circuits needed to be considered to create a manageable one way circuit. The footprint had to be necessarily bigger so that within stations all participants could maintain the two metre distancing from each other. This change led to a need for additional distance between stations which we factored in to our planning. Usually our OSCE consists of two minutes of moving and reading time then an eight minute station (in line with the national examination) however with the more widely spread circuit and the need to clean down the stations between students we introduced an additional three minute gap between each station (for picture examples see Supplementary File 1). This was monitored by the invigilators and if all participants were ready for the next students earlier then the reading time was signalled to start. Due to this increased time between stations, the decision was taken to remove any rest stations from the circuit as it was felt that this would unnecessarily prolong the process. Feedback from students was that they felt that over ten stations there was no excess fatigue due to this however an increase in the number of stations would have necessitated a rest station. Detailed plans were provided to invigilators regarding the movement phases to ensure students could be prevented from coming into closer than two metre contact with each other. In addition, it was determined safest practice to keep any doors open that were needed for the flow of the circuit while the examination was in progress to reduce disease spread via the door handles.

## Staffing the examination

Experienced examiners were recruited from within the faculty. Whilst normally we would recruit additional assessors in case of any late changes the decision was taken that the course leads, both experienced examiners, could step in should there be such an issue to prevent any unnecessary additional people on circuit. No expert patients were used for the circuit due to the higher risk that they represented and so actors were recruited for the stations. The actors were chosen with consideration of the case demographics so as not to add a potential distractor that could affect student performance. In order to minimise the risk should an actor be unable to attend on the day all actors were asked to prepare for two stations allowing additional flexibility if needed. Stations were identified where, if needs be, course leads could step in to support in the absence of an actor.

In order to aid the quality assurance and reduce footfall on the circuit we set up video feeds from each station using iPads. We created a video conference space using Blackboard Collaborate (the Universities virtual learning environment) for each station. The quality assurance team and our external examiner were provided with links to each station feed allowing them to provide external scrutiny without having to attend in person and the ability to observe quickly any station where concern may have arisen. One of the only issues encountered on the day was that one of these iPads occasionally dropped off the wireless network however we were able to re-establish connection when this occurred with minimal interruption. In addition, depending on the placement of the ipads within the station it could be difficult to fully capture both audio and visual input from both patient and student which is something we will need to consider for the future.

WhatsApp groups were created to facilitate conversation between key groups. One was created for the invigilators, one for the assessors and one for the quality assurance team. The course and OSCE leads were members of all three groups to act as coordinators of the process. The use of these groups allowed any issues to be rapidly raised and acted upon without it becoming “white noise” had everyone been in a single group.

## Prior to the exam day

Prior to the day of the examination recorded briefings were sent to examiners and actors in order to avoid the need to undertake a group briefing with an offer to undertake one-to-one conversations online should there be any queries. All of those participating on the day were reminded of current national guidance on social distancing, use of face coverings on public transport if using that to attend, and undertook a short training session online mandated by the University itself. Participants were also advised that should they have had Covid19 symptoms in the seven days prior to the examination, or been exposed to a known Covid19 positive individual in the previous 14 days, they must not attend and inform us at the earliest convenience.

Students were sent an extraordinary assessment document setting out how the examination would differ from the process set out in existing course regulations and were required to send back a signed declaration that they had understood. This included the process for students for whom a “not assessed” decision may be applied due to potential Covid infection. Had this been needed we could have undertaken a further summative OSCE reducing the circuit size but utilising the same principles. Had a further OSCE been need for resits this would have been undertaken purely online with supporting evidence of competence from previous assessments.

All participants were advised to bring their own food and drink as no one was allowed to leave the premises and providing the usual buffet was considered inappropriate in the circumstances. Fridge space was made available if required.

The day in advance of the examination a small group went into the building in order to set the circuit up including markings on the floor to ensure students could follow an effective one way system on the circuit, had clear visual cues to maintain appropriate social distancing, and to create signposting throughout the building in order to maximise participant safety on the day. Examples of the circuit can be seen in Supplementary File 1. Setting up involved ensuring hand sanitiser was available at the building entrance, on every station and in the holding rooms as well as ensuring appropriate cleaning materials were available in each station. Defined access and egress routes were put in place around the circuit and building to ensure everyone participating would be able to maintain safety. The circuit was re-cleaned on the morning of the OSCE with careful consideration of frequent touch points such as door handles prior to the start of the circuit.

## On the exam day

On arrival at the exam venue all participants had their temperature checked by one of the exam leads who wore protective equipment in line with Public Health England Guidance for primary care ( Public Health England, 2020). A guide tympanic temperature of 37.8°C was used as a cut off ( Sund-Levander, Forsberg and Wahren, 2002) and anyone with a temperature above this was not permitted to enter the building. Provision had been made for any student with a raised temperature to receive automatic extenuating circumstances for the examination for which a “not-assessed” decision would be applied. Fortunately this did not apply to any students. Once in the building students were directed to their holding room which was set out to ensure social distancing whilst actors and examiners were directed straight to their stations where they remained except at lunchtime when a communal space, marked for distancing was utilised.

Students were separated into a morning and afternoon group. The morning group remained in their holding room until the afternoon group had been registered in a separate holding room. This was to prevent mixing of the two students groups as is our normal practice. The circuit design ensured that those students arriving in the afternoon never entered any area being utilised by the examination until their circuit started.

In our OSCE paper and pens are usually available outside each station for students to make notes on. Instead of this students had been instructed to bring their own should they wish it and a notebook was permitted rather than loose sheets but students had to demonstrate to invigilators that it was blank prior to the start of the assessment.

The marking was all done using iPad tablet devices with wipe clean covers and software that produced immediate results removing any need for handling of paper from examiners and in the results analysis. All tablets were cleaned prior to the morning circuit starting and an entirely new set were used for the afternoon circuit ensuring that they would be fully charged and therefore little risk of battery failure and necessitating a swap out. Paper copies of the mark sheets were also available in each station in case of IT failure as an emergency back-up per normal practice.

Invigilators wore face coverings while the circuit was in progress to mitigate the difficulty maintaining the two metre distancing as they coordinated students moving between stations. All actors and assessors were required to wear face coverings when moving around the building however these could be removed once distanced on station. The students and actors had the option of wearing a face shield while the station was in progress if they wished. Participants were asked to wash or sanitise their hands before and after toilet use and coming into direct contact with surfaces such as door handles.

At the end of the day students were taken off the circuit to their holding room whilst actors and invigilators were taken off the circuit by following the one way system around to the exit. Invigilators and actors were allowed to leave first and once they were clear the students were permitted to leave. This process ensured that everyone could maintain social distancing. The OSCE team then undertook the circuit close down.

## Subsequent formative OSCE

Following this OSCE it was decided that students would benefit from additional OSCE practice through a formative examination. Prior to undertaking this we had guidance to changes in the National examination, specifically patients all being virtual on screen and not in the same room. Due to this we converted two stations to run in this format. The nature of the building was such that these were via a wireless network and on one occasion we lost the patient feed due to a poor connection although were quickly able to re-establish it. The National examination used wired connections which proved very stable and, where possible, we would recommend this. If not we found that changing the stations such that the wireless was nearer the main hub in the building solved the issue. Feedback following the national examination showed that students felt they had been well prepared through this process.

## Conclusion

The Covid19 pandemic led to some specific challenges that needed to be overcome in order to deliver a face to face OSCE. The planning and mitigations put in place enabled this to occur with minimal risk to participants whilst delivering a defensible examination permitting students to be assessed at a “shows how” level of Millers pyramid ( Miller, 1990). With the search for a vaccine still ongoing and therefore the likely need to adapt assessment for some time we believe that this work can act as a guide for others seeking to undertake their own summative clinical examinations and enable them to learn from our experiences.

## Take Home Messages

• The building infrastructure is important to ensure that a successful OSCE can be run. Ensure all necessary checks have been carried out on previously closed buildings and that the circuit can be set up with appropriate spacing.
• Follow the guidance in place in your country for Covid Secure workplaces to ensure that you meet all necessary standards to protect participants.
• Preparation beforehand with good marking of one way systems, consideration of access and egress, and briefings undertaken virtually can reduce face to face contact and therefore minimise risk for all participants.
• It is recommended to mentally and physically walk through every detail from the point of arrival through the circuit and to the point of exit to ensure that all potential areas of disease spread have been addressed and mitigated.
• Some changes made out of necessity will be continued going forwards - specifically the video streaming of stations and the WhatsApp group communication.

## Notes On Contributors

**Claire Darling-Pomranz** is a University Clinical Teacher within the Academic Unit of Primary Medical Care at the University of Sheffield and a practising Physician Associate.

**Dr James Gray** is Senior University Teacher at the University of Sheffield within the Academic Unit of Primary Medical Care and a practising General Practitioner. ORCID ID: https://orcid.org/0000-0003-0821-5789

**Charlotte Watson** is Administration Manager at the University of Sheffield within the Academic Unit of Primary Medical Care.
