# Supplementary material for: Undertaking a face to face Summative OSCE assessment during the COVID-19 Pandemic - a descriptive narrative
Source: MedEdPublish (2016). 2021 Mar 17;9:244. Originally published 2020 Oct 29. [Version 2] doi: 10.15694/mep.2020.000244.2 (PMC10697526; doi:10.15694/mep.2020.000244.2)
Supplement: Peer review history

# Reviewer report: Reviewer response for version 2

**DOI:** 10.21956/mep.20281.r31487

**Published:** 2021-06-13

**Authors:** Ken Masters

**Affiliations:** 1 Sultan Qaboos University

This review has been migrated. The reviewer awarded 4 stars out of 5 This is Version 2 of an already-strong article, and the authors have addressed the Version 1 reviewers’ concerns, and cleared up small issues that needed clarification. Although every situation is different, the lessons learned and explained in the paper will greatly assist those wishing to undertake a face-to-face summative OSCE assessment during the Covid-19 (and other) pandemic.

Reviewer Expertise:

NA

No decision status is available

# Reviewer report: Reviewer response for version 2

**DOI:** 10.21956/mep.20281.r31488

**Published:** 2021-06-04

**Authors:** Barbara Jennings

**Affiliations:** 1 Norwich Medical School

This review has been migrated. The reviewer awarded 4 stars out of 5 Thank you for submitting your revised article in response to the reviewer comments linked to version 1; https://www.mededpublish.org/manuscripts/3465. Your author revision-notes section is a useful summary of the changes and the extra details included in this version 2 have enhanced the article.Thank you to the authors for documenting your local experiences delivering a F2F OSCE for the Physician Associate cohort during the pandemic and for citing the safety guidelines that were followed.

Reviewer Expertise:

NA

No decision status is available

# Reviewer report: Reviewer response for version 1

**DOI:** 10.21956/mep.19141.r27670

**Published:** 2020-11-06

**Authors:** P Ravi Shankar

**Affiliations:** 1 American International Medical University

This review has been migrated. The reviewer awarded 4 stars out of 5 This is an interesting paper and as the pandemic persists on-site OSCEs and clinical exams will need to be conducted. The authors have described the process well. My queries are about reducing the number of stations from 14 to 10. Will that affect the validity of the OSCE? Was this examined? The other is about the number of students who participated in the OSCE. Are there any modifications to the national OSCE? What exactly is the role of a physician associate? Also, the rest station was removed. Did the examines feel that this made the OSCE more strenuous? The article will be useful to all educators planning OSCEs as part of student assessment.

Reviewer Expertise:

NA

No decision status is available

# Reviewer report: Reviewer response for version 1

**DOI:** 10.21956/mep.19141.r27669

**Published:** 2020-10-30

**Authors:** Johnny Lyon Maris

**Affiliations:** 1 Southampton GP Education Unit

This review has been migrated. The reviewer awarded 4 stars out of 5 Thank you for sharing this descriptive piece, as this is the only way we are going to learn as a community. I applaud your resolve to go ahead, but wonder if you had exhausted a virtual approach with actors on a digital platform or an alternative form of assessment - recorded live consultations? I would have liked to see your rationale for reducing from 14 stations to 10, how that decision was made and the psychometrics behind the decision. You do not mention the numbers of candidates and how you dealt with those unable to attend (due to isolation/shielding) or those turned away on the day for health reasons. These queries are not criticisms but genuine interest as all health professions are trying to work out how to maintain standards and assess differently.

Reviewer Expertise:

NA

No decision status is available
